# Novel Parallelized Electroporation by Electrostatic Manipulation of a Water-in-Oil Droplet as a Microreactor

**DOI:** 10.1371/journal.pone.0144254

**Published:** 2015-12-09

**Authors:** Hirofumi Kurita, Shota Takahashi, Atsushi Asada, Minako Matsuo, Kenta Kishikawa, Akira Mizuno, Rika Numano

**Affiliations:** 1 Department of Environmental and Life Sciences, Toyohashi University of Technology, Toyohashi, Aichi, Japan; 2 Electronics-Inspired Interdisciplinary Research Institute (EIIRIS), Toyohashi University of Technology, Toyohashi, Aichi, Japan; University Paul Sabatier, FRANCE

## Abstract

Electroporation is the most widely used transfection method for delivery of cell-impermeable molecules into cells. We developed a novel gene transfection method, water-in-oil (W/O) droplet electroporation, using dielectric oil and an aqueous droplet containing mammalian cells and transgene DNA. When a liquid droplet suspended between a pair of electrodes in dielectric oil is exposed to a DC electric field, the droplet moves between the pair of electrodes periodically and droplet deformation occurs under the intense DC electric field. During electrostatic manipulation of the droplet, the local intense electric field and instantaneous short circuit via the droplet due to droplet deformation facilitate gene transfection. This method has several advantages over conventional transfection techniques, including co-transfection of multiple transgene DNAs into even as few as 10^3^ cells, transfection into differentiated neural cells, and the capable establishment of stable cell lines. In addition, there have been improvements in W/O droplet electroporation electrodes for disposable 96-well plates making them suitable for concurrent performance without thermal loading by a DC electric field. This technique will lead to the development of cell transfection methods for novel regenerative medicine and gene therapy.

## Introduction

A variety of cell-impermeable molecules, such as DNA, RNA, proteins, antibodies, and dyes, have been delivered into cells in a wide range of fields, such as life science, medicine, pharmacy, and agriculture. Transfection, which is a fundamental technique used to deliver nucleic acids into mammalian cells, is widely used for experimental and therapeutic purposes. For example, viral vector, electroporation, lipofection, particle gun, and sonoporation methods have been used for transfection. These methods have both advantages and disadvantages with regard to delivery efficiency, viability, running costs, etc. Therefore, the development of safer, more effective, and low-cost novel transfection methods is required. Electroporation generates transient membrane pores when the transmembrane potential exceeds a critical threshold value, altering the permeability of the membrane by application of an external electric field. Electroporation offers several important advantages compared to viral, chemical, and liposome-based transfection methods, including high transfection efficiency, reduced safety concerns, simple operation, and no restriction on cell type and exogenous material properties [1, 2]. However, most commercial electroporation-based transfection methods require the use of specialized pulse generators to produce short electrical pulses at high voltage. Therefore, novel electroporation techniques based on simple and low-cost equipment are required.

To resolve these issues, microfluidic-based electroporation systems have been developed. These systems provide flow-through electroporation that can be used for high-throughput transfection. In addition, by applying microelectronic pattern techniques, the distances between the electrodes in the microchips can be made so short that relatively low potential differences are sufficient to yield sufficient electric field strengths [3–9]. Moreover, use of water-in-oil (W/O) droplets in microfluidic systems has been investigated [10–13]. In such systems, each droplet can be considered as a microreactor with a tiny volume, thus reducing reagent consumption and achieving rapid mixing.

In our p study, we demonstrated a quite different electroporation method based on electrostatic manipulation of W/O droplets in a DC electric field. There have been a number of recent reports regarding electrostatic charging and subsequent manipulation of W/O droplets [14–21]. When an aqueous droplet is suspended in a dielectric liquid, such as oil, it can be used as a small reactor and moved between a pair of electrodes with application of a DC electric field. This droplet motion is brought about as follows. First, a droplet is carried to one electrode by Coulomb force, possibly due to electrostatic induction. When the droplet makes contact with the electrode, the droplet acquires a charge with the same polarity as the electrode. The droplet then moves to the other electrode and the same process occurs repeatedly. During this periodic motion, a local intense electric field is applied to the droplet in a very short time when it comes into contact with the electrode. Im *et al*. experimentally investigated the effects of high electric fields on the viability and proliferation of living mammalian cells inside charged droplets under electrostatic actuation [22]. Their results indicated no noticeable influence of the electric field and silicone oil on cell viability and proliferation. During the periodic bouncing motion of a droplet between two electrodes, the local intense electric field is useful for gene transfection. Furthermore, a more intense electric field can induce droplet deformation, leading to instantaneous short circuiting via the droplet. Our previous study showed that *Escherichia coli* was transformed by electrostatic actuation of the droplet containing the bacterial cells and plasmid DNA [23]. Here, W/O droplet electroporation also enabled efficient transfection of various mammalian cells with high cell viability.

## Materials and Methods

### Construction of plasmid DNA and cell preparation

The Venus improved yellow fluorescent protein (YFP) plasmid was kindly provided by Prof. A. Miyawaki (Brain Science Institute, RIKEN) [24]. For cotransfection analysis of both green fluorescent protein (GFP) and red fluorescent protein (mCherry or TagRFP) expression, fluorescent protein-expressing plasmid were purchased from Clontech Laboratories, Inc.. The HEK293 cell line (from human embryonic kidney cells) and TIG-117 and 108 fibroblasts cell lines (from human skin fibroblasts established from human subjects aged 81 and 12 years old) were distributed from JCRB Cellbank (Health Science Research Resources Bank) were used for gene transfection. The suprachiasmatic nucleus (SCN) neuronal cell line RS182, which was derived from the SCN of the offspring of crossing homozygous *Per1*::luc [25] and heterozygous tsA58 transgenic rats [26], was a kind gift from Prof. H. Tei (Kanazawa University). RS182 cells were incubated for at least 5 days at 33°C for proliferation in 10% FBS/DMEM (Wako) and changed to neurobasal medium (Invitrogen) supplemented with 2% B27 (Invitrogen) and antibiotics (Invitrogen) at 39°C. These cells were plated at 1–5×10^5^ cells/mL on culture plates for 24–48 hours and transfected 9×10^3^−6×10^4^ cells with 0.25–2 μg of expression plasmid DNA in 3-μL droplets with phosphate buffered saline (PBS) in all experiments. Mouse hippocampus cells derived from day-16 embryo were purchased from Sumitomo Bakelite co..

### Transfection by water-in-oil droplet actuation

**Fig 1:**
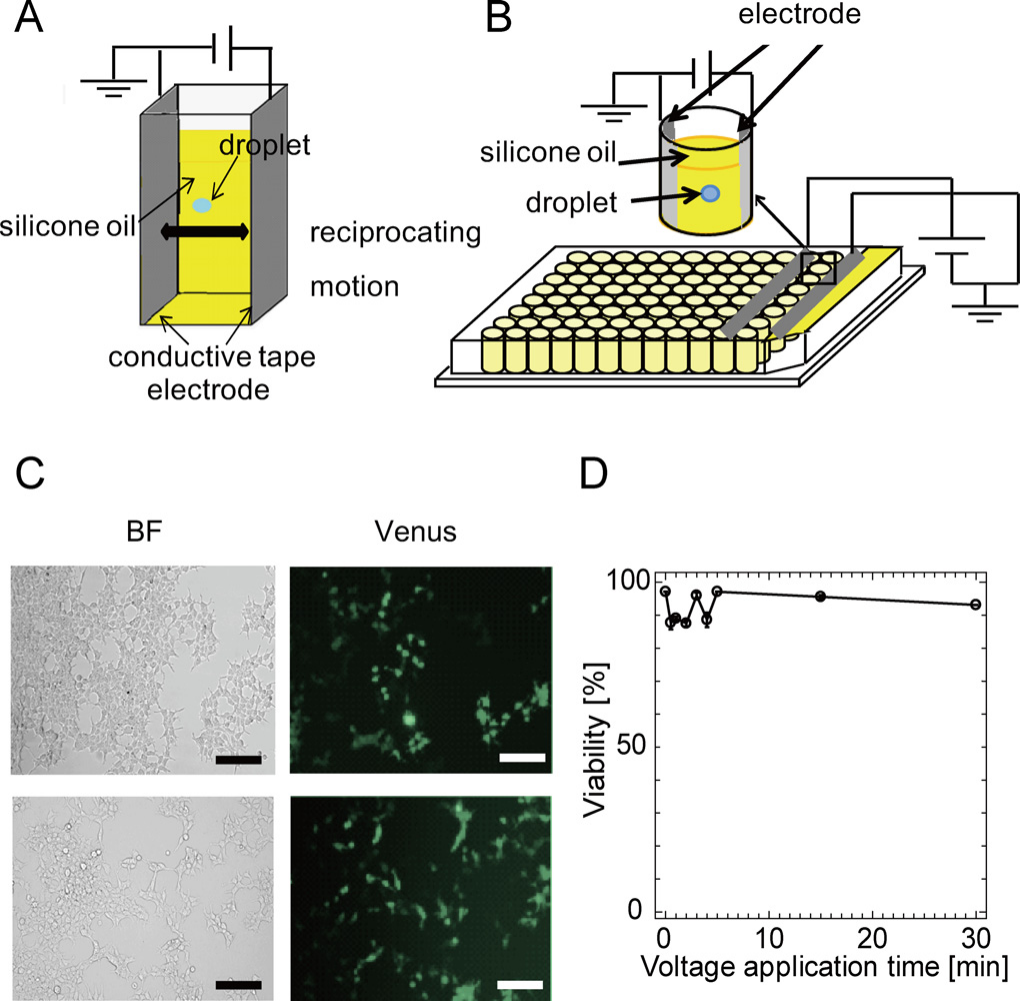
The droplet actuation device and cells transfected by the W/O droplet electroporation method. (A), (B) Two pieces of conductive tape were set parallel on a single cuvette or 8 wells in a line for 96-well plastic microwell plates. The droplet continued to bounce between the edges of the two electrodes. One was the ground electrode, and the other was the high-voltage electrode. Images of droplets bouncing between the anode and cathode in each well are shown. Many cells in droplets can be transfected simultaneously. (C) The upper row : Bright-field (BF), fluorescence, and merge images of HEK293 cells 24 hours after transfection by W/O droplet electroporation. Cells were examined at 24 hours after transfection to evaluate the expression of fluorescent protein (Venus). Scale bars, 100 μm. The lower row : BF, fluorescence, and merge images of HEK293 cells 24 hours after transfection by lipofection. (D) Variation of cell viability with time of droplet actuation determined by trypan blue staining. All experiments were performed at least twice.

Fig 1A shows the droplet actuation device. Pieces of conductive tape, one of which was the ground electrode and the other was the high-voltage electrode, were set in a single cuvette and multi-microwell plates. The cuvette or wells of the 96-well plates were then filled with 0.2 mL of silicone oil (KF96-1, 100 cSt kinematic viscosity, 2.74 dielectric constant, 965 kg/m^3^ density; Shin-Etsu Chemical Co.) without any surfactant. The 3-μL droplet with suspended cells (3000–10000 cells/droplet) and plasmid DNA (0.25–2 μg) was dispensed in silicone oil and a high voltage was then supplied to move the droplet using a DC high-voltage power supply (HAR-30R10 or HJPM 5R1.2; Matsusada Precision Inc.). A high voltage was applied for 2–5 minutes to move the droplet. After applying the DC electric field, the droplet was recovered and transferred to culture wells with 0.1–1 mL of culture medium in a glass-bottom dish plate for inverted microscope imaging (IWAKI). Stable cell lines transfected Venus plasmid by W/O droplet electroporation were established for one month and frozen in stock solution CELLBANKER (Takara). Three of the colonies were expanded into the culture medium and cultured for one more month. Fibroblast and hippocampus neuronal cells in 3-μL droplet were doubly transfected with both 0.4 μg of Venus and 0.4 μg of mCherry plasmid DNA in identical 3-μL droplets with PBS and incubated for 3 day-2 week in DMEM or neurobasal medium at 37°C (*n* = 3–8). HEK293 cells in 3-μL droplet were doubly transfected with both 0.4 μg of GFP and 0.4 μg of TagRFP plasmid DNA in identical 3-μL droplets with PBS and incubated for 3 day-2 week in DMEM medium at 37°C (*n* = 4).

The attached cells (HEK293, fibroblast and hippocampus neuronal cells and so on) were cultured for more than two days after W/O droplet electroporation and changed to a new culture medium every few days. The dead cells in the culture medium were washed out at the changing of the medium. All experiments were performed two to four times.

### Imaging

Cells were observed 24 hours after transfection to evaluate the expression of fluorescent protein (Venus) in a glass-bottom dish plate for inverted microscope imaging (IWAKI). Both bright-field and fluorescence images of cells were captured using an inverted fluorescence microscope (TE-2000U; Nikon) equipped with a 100× oil immersion objective (PlanApo; Nikon) and a digital camera (D3100; Nikon). Cells were also observed using a fluorescence microscope (Olympus; IX81) equipped with an objective lens (Olympus, UFlanSApo 10× and PlanApoN 60×), a blue LED for excitation (BMC), and a CCD camera (ImagEM; Hamamatsu Photonics) overnight after transfection. Imaging analysis was performed using MetaMorph software (Molecular Devices) and NIS-Elements (Nikon). The intensities of Venus and mCherry fluorescence in cells were observed by alternating excitation at 488 nm and 590 nm with emission at 510 nm and 610 nm, respectively. At least four cell images were taken from one dish.

## Results

### Development of water-in-oil (W/O) droplet electroporation

Electroporation is the most widely used physical method for delivery of cell-impermeable molecules into mammalian cells due to its versatility and simplicity. We developed a novel gene transfection method, water-in-oil (W/O) droplet electroporation, using dielectric oil and an aqueous droplet containing mammalian cells and transgene DNA.

Initially, the prototype W/O droplet electroporation system was constructed using a 3-mL cuvette and two pieces of conductive tape (Fig 1A and 1B). The distance between the electrodes was about 10 mm, so the applied electric field intensity was approximately > 3 kV/cm. Fig 1B shows the droplet actuation device with improved W/O droplet electroporation electrodes for disposable 96-well plates. Two pieces of conductive tape were set parallel on 8 wells of 96-well plastic microwell plates with silicone oil. An aqueous droplet containing mammalian cells and foreign plasmid DNA bounces between a pair of electrodes by Coulomb force in dielectric oil with application of a DC electric field.

Aliquots of 3 μL of the prepared cell suspension containing 3000–10000 cells and plasmid DNA (0.25–2 μg) were added to the oil, and a DC high voltage (2.5 kV for 96-well plates) was applied to move the droplet for 2–5 minutes. After applying the DC electric field, the droplet was recovered and applied to 0.1–1 mL of culture medium. The droplet acquired charge and moved to the electrode with the opposite polarity. The droplet moved toward the edge of the electrode during the bouncing motion, due to the short electrode spacing of 1 cm and 5 mm between the electrodes (S1 Movie). Finally, the droplet continued to bounce between the edges of the two electrodes. Fig 1C shows bright-field and fluorescence images of HEK293 cells (human embryonic kidney cells) 24 hours after electroporation. Fig 1D shows similar images of HEK293 cells but transfected by lipofection (Lipofectamine2000; Life Technologies). In both figures, Venus-expressing HEK293 cells were observed after transfection. These observations suggested that our novel transfection method yields transfected cells and is thus comparable to lipofection. Fig 1E shows cell viability after electroporation plotted as a function of voltage application time. The cell viability was counted by staining cells with 1 μg/mL of trypan blue (Invitrogen) in PBS, and was > 85% for each period. 15% in Fig 1D indicates cell death ratio soon after W/O electroporation with about 2 kV by the trypan blue assay. Therefore, the use of silicone oil and a high electric field had little effect on the viability of HEK293 cells in W/O droplets under conditions of droplet actuation as observed previously in *E*. *coli* [23]

### Suitable conditions for W/O droplet electroporation

**Fig 2:**
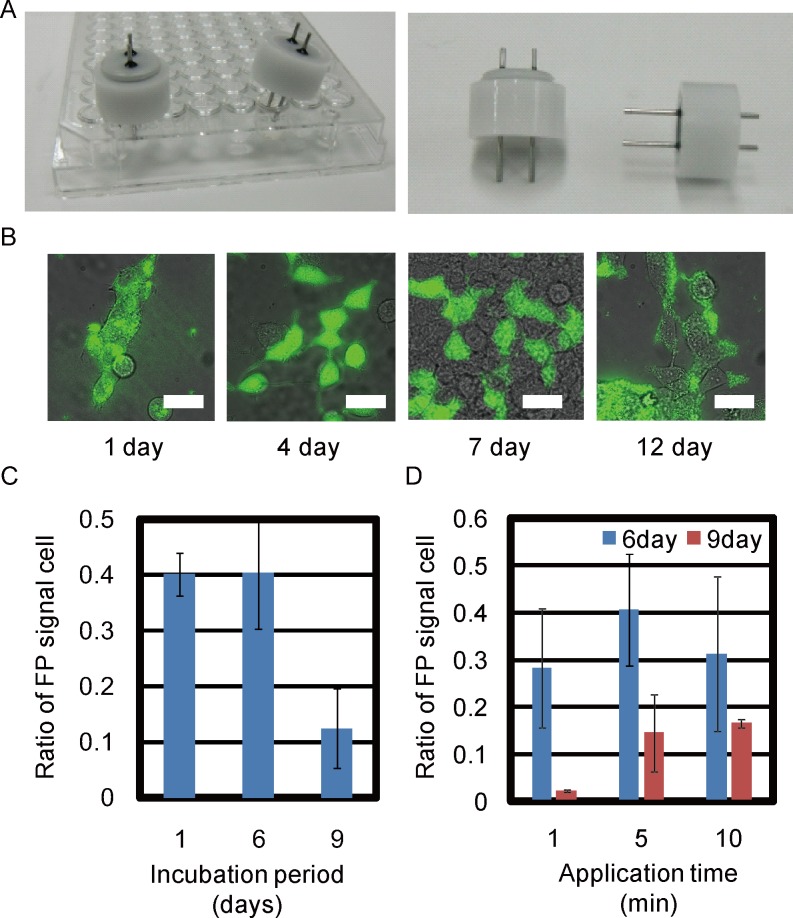
Screening of suitable conditions for W/O droplet electroporation. (A) The pair of pin electrodes for single well of 96multi-well plate. (B) Merge image between bright-field (BF) and fluorescence images of HEK cells 1, 4, 7, and 12 days after W/O droplet electroporation. All scale bars, 30 μm. (C) HEK293 cells transfected with Venus fluorescent plasmid 1, 6, and 9 days after W/O droplet electroporation. Transfection efficiency was compared with 5-minute application time. (D) HEK293 cells transfected with Venus plasmid with application times of 1, 5, and 10 minutes 6 and 9 days after electroporation.

Eight wells of 96-well plastic microwell plates in Fig 1B showed unequal bouncing motion of the droplet in each well. The new pairs of pin electrodes for each well in 96-well plates were manufactured with a spacing of 6 mm (Nepa Gene) (Fig 2A). Venus plasmid could be transfected into various cells by W/O droplet electroporation. The transfection efficiency was compared 1) between various incubation periods following 5-minute application and 2) between various W/O droplet electroporation application times. HEK293 cells were transfected with Venus vectors in 96-well plates for 5 minutes at 2 kV (± 0.2 kV). The Venus signal was examined by fluorescence microscopy (Fig 2B). In this experiment, many healthy cells were observed with stronger fluorescent signals compared with autofluorescence and sufficient signal intensity compared with cells showing maximal signals in the same image. The fluorescence intensity of each cell was divided by the corresponding cell area (mean intensity) and compared with autofluorescence calculated from control cells without W/O droplet electroporation (*n*≥10) (S1 Fig, S2 Fig).

The transfection efficiency was calculated as the number of cells with positive signals divided by the total number of cells in the same images (Fig 2C and 2D). Dead cells with autofluorescence signals were not counted in the images. The transfection efficiency of Venus plasmid in HEK293 cells was compared after various incubation periods (Fig 2C) or various application times (Fig 2D) after W/O droplet electroporation with electrodes in 96-well plates at 2 kV (± 0.2 kV). The total number of all cells in one image is between 11–150 after W/O electroporation.

Venus signals of each cell after electroporation for 5 minutes were counted among all cells in fluorescence microscopy images using either NIS-Elements (Nikon) or MetaMorph software (Molecular Devices) on various days after W/O droplet electroporation. Venus fluorescence signals were examined 1, 4, 7, and 12 days after transfection (S2 Fig). The mean fluorescence signal remained significantly high compared with negative controls without electroporation (*p* < 0.0001). The transfection efficiencies determined from Venus fluorescence signals in HEK293 cells were 40.2%, 40.4%, and 12.4% (averages of 5, 77, and 215 cells per image, respectively) 1, 6, and 9 days after W/O droplet electroporation, respectively (Fig 2C).

The transfection efficiency was compared between various application times 6 days after electroporation. Positive Venus fluorescence signals were observed in 28.3%, 40.7%, and 31.2% of HEK293 cells transfected for 1, 5, and 10 minutes, respectively. The transfection efficiencies were 2.2%, 14.5%, and 16.6% for 1, 5, and 10 minutes 9 days after electroporation (Fig 2D). These results indicated that transient transfection efficiency was not different with application of W/O droplet electroporation for more than 5 minutes. Although 1-minute application time was not sufficient to maintain transgene expression over 9 days, an application time of 5 minutes was sufficient to achieve 40% transfection efficiency (Fig 2D). Subsequent experiments involving transfection by W/O droplet electroporation were performed for 3–5 minutes, because lethality and transient transfection efficiency were not different between 3 and 5 minutes.

### Development of water-in-oil (W/O) droplet electroporation

**Fig 3:**
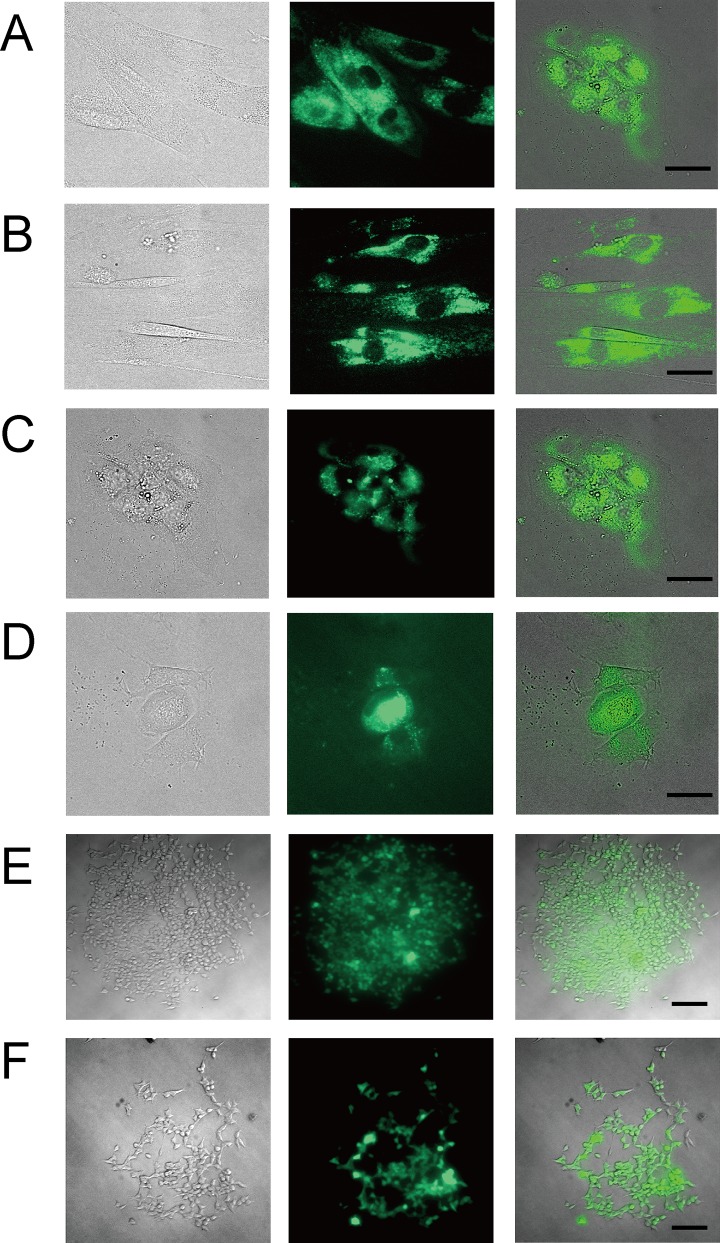
Fibroblast, SCN neural cells and HEK293 cells transfected Venus plasmid by W/O droplet electroporation. (A) Bright field, fluorescence and merge images of fibroblast cells from a subject aged 81 years old one day after electroporation. (B) 7 days after W/O droplet electroporation. (C) Bright field, fluorescence and merge images of SCN neural cells three days after W/O droplet electroporation. SCN cells had been differentiated to neural cells by incubation at higher culture temperature [26]. (D) Twelve days after W/O droplet electroporation. All scale bars (A-D), 30 μm. (E) Bright field, fluorescence and merge images of HEK293 cells colonies were picked up and cultured for one month after W/O droplet electroporation. (F) Bright field, fluorescence and merge images of HEK293 cells colonies were cultured though frozen stock for a total of more than two months after W/O droplet electroporation. scale bars (D-E), 100 μm.

Transgene DNA could also be transfected into small numbers of cells (2000 cells) of fibroblast cell lines derived from human subjects aged 81 and 12 years old with high efficiency (Fig 3A and 3B). These observations suggested that even small numbers (1000–3000) of many kinds of cells could be transfected by W/O droplet electroporation. The W/O droplet electroporation method has several advantages compared with other transfection techniques reported previously, including simultaneous transfection of various types of DNA into even as few as 1000 cells, transfection into differentiated SCN neural cells, and the establishment of stable cell lines. Many transient and some stable fibroblast cells maintained Venus fluorescence signals for 1 (Fig 3A) and 7 days (Fig 3B), for 3 (Fig 3C) and 12 days (Fig 3D) after transfection with Venus plasmids by W/O droplet electroporation. W/O droplet electroporation successfully introduced transgene DNA into differentiated SCN neural cells, which were estimated to be neural cells based on Nestin expression and morphology [26]. Mouse hippocampus primary neuronal cultured cells dispersed from embryonic hippocampus tissue by papain solution and grown in primary culture in neurobasal medium for 12 days (Sumitomo Bakelite Co.) could be transfected with Venus plasmid and red fluorescence plasmid by W/O droplet electroporation (S3 Fig). Therefore, we think that a stable cell line may be established because the expression of fluorescent transgene was maintained from one week to one month and because the fluorescent signal could be observed in pickup colony total about two months after electroporation through freezing stock (Fig 3D–3F). It indicates the successful establishment of double stable cell lines.

### Application of W/O droplet electroporation to co-transfection of multiple types of DNA and stable transfection

**Fig 4:**
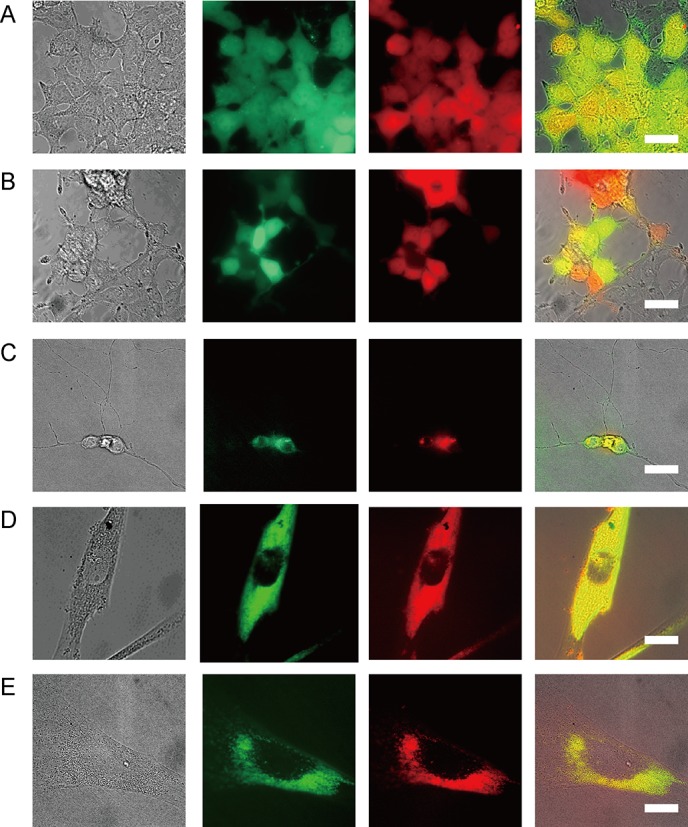
Double and stable transfected cells by W/O droplet electroporation. (A), (B) Transgene plasmid DNAs of two kinds of fluorescent protein: green fluorescent protein (GFP) and Tag-RFP (red fluorescent protein), were successfully double transfected into HEK293 cells by W/O droplet electroporation for 3 minutes. Bright-field image and fluorescent GFP or RFP image of HEK293 cells (A) 5 days (71% of cells maintained GFP and 71% of cells maintained TagRFP signal) and (B) 8 days (41% of cells maintained GFP and 37% of cells maintained TagRFP signal) after W/O droplet electroporation. Scale bars, 30 μm. (C) Transgene plasmid DNAs of Venus and mCherry (red FP) were successfully double transfected into hippocampus primary neural cell lines by W/O droplet electroporation for 5 minutes. Scale bars, 30 μm. (D), (E) Bright-field image and fluorescent image of Venus and mCherry expression in human fibroblast cells (D) 2 days and (E) 10 days after W/O droplet electroporation. Scale bars, 30 μm.

The transfection efficiency of co-transfection in HEK293 cells (two kinds of fluorescent protein plasmid (GFP and TagRFP) with green and red fluorescent signals) was calculated after various incubation periods after W/O droplet electroporation or for various applications times in 96-well plates at 1.8–2.2 kV for 3–5 minutes. The number of cells expressing both fluorescent proteins was counted among cells showing some fluorescent signal as shown in Fig 4. 30 cells with both fluorescences among 42 cells in one image (Fig 4A). 10 cells with both fluorescences among 27 cells in one image (Fig 4B). The cotransfection efficiencies of both GFP and TagRFP fluorescent plasmid DNA were over 70% five days after W/O droplet electroporation (Fig 4A) and were more than 37% eight days after W/O droplet electroporation (Fig 4B).

We confirmed that double transgenes could be simultaneously transfected into fibroblast cells within 5 minutes by inclusion of both Venus and mCherry transgenes with yellow and red fluorescent signals, respectively, in the droplets for electroporation (Fig 4C–4E). In addition, it was confirmed that stable cell lines could be established by W/O droplet electroporation. Strikingly, many fibroblast cells maintained double fluorescence signals from Venus and mCherry expression vectors for 2 days (Fig 4D), 10 days (Fig 4E), and even 1 month after transfection with Venus and mCherry plasmids by W/O droplet electroporation.

### Concurrent performance of W/O droplet electroporation electrodes for multiwell plates

**Fig 5:**
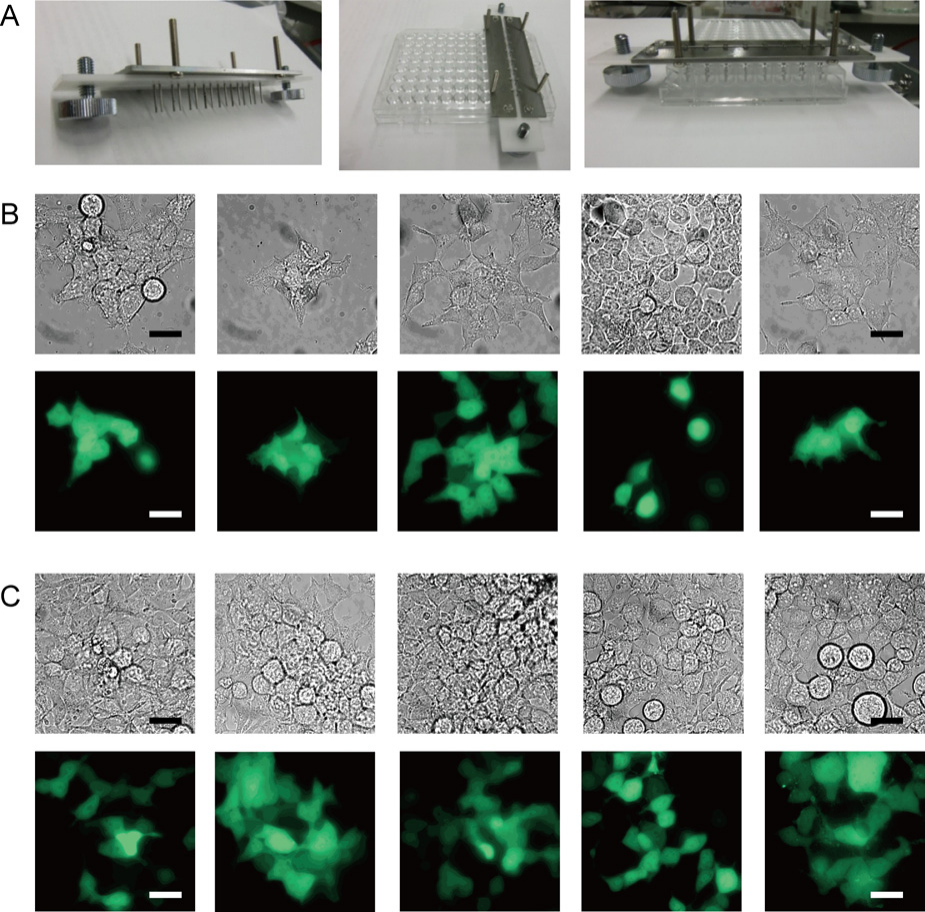
Image of the parallel W/O droplet electroporation electrode for the 8-well string of disposable 96-well plates. (A) Conductive electrodes were produced and set on an 8-well string in 96-well microwell plates for W/O droplet electroporation. (B), (C) Venus plasmid was transfected into some HEK293 cells using 8-well W/O droplet electroporation electrodes (Nepa Gene) for 96-well plates at 1.8–2.2 kV. Venus fluorescence signals were observed after incubation in 5 of 8 wells for 4 and 7 days following electroporation. Scale bars, 20 μm.

The number of bouncing droplets may be positively correlated with transfection efficiency, as the intensity of the electric field would be highest when applied to the surface of the cell membrane at the electrodes during droplet bouncing. Therefore, a shorter distance between the edges of the electrodes and higher frequency of droplet bouncing would be associated with greater transfection efficiency. Fig 5A shows the droplet actuation device for 8 wells in a line of disposable 96-well plates with improved W/O droplet electroporation with pairs of pin electrodes (Nepa Gene). Venus plasmid was transfected into some samples of HEK293 cells using 8-well electrodes at 2 kV (±0.2 kV). Venus fluorescence signals were maintained in 5 of 8 wells 4 and 7 days after W/O droplet electroporation (Fig 5B and 5C).

## Discussion

Conventional electroporation relies on the application of sufficiently strong electric pulses to cell suspensions to promote the formation of transient pores in the cell membrane. The transport of charged macromolecules induced by the electric field is also considered to be an important factor in electroporation, although a detailed understanding of the effects of electric fields on the transport of macromolecules into cells remains elusive. As in the W/O droplet electroporation method investigated here, It is assumed that the formation of transient pores in the cell membrane could be induced by a pulsed electric field. From the principle of droplet bouncing, the pulsed electric field could be applied when a droplet makes contact with the electrode and acquires a charge with same polarity as the electrode. In addition, electrical charges at the interface between the water and oil phases could form a local electric field between the interface and negative charges on both DNA and the cell membrane, which could affect the transport of macromolecules. Although the transfection efficiency of electroporation depends on various experimental conditions, such as cell lines, confluency of cells, and pulse parameters, conventional electroporation.

To explain the mechanism of the transfection, the transient pore formation of cell membrane is one of the essential processes. Therefore, we tried to show the transient pore formation on the cell membrane. The uptake of fluorescent dyes which do not enter the cell membrane of live cells is commonly carried out to detect permeabilization by electroporation [27–29]. Here, DNA staining dye, YO-PRO-1 (Life technologies), was used which is practically non-fluorescent until entering the cell and binding to nucleic acids. The fluorescent intensities after the transfection had clearly increased (S5 Fig). Therefore, transient pores were formed in the cell membrane by our method. Moreover, that was because stable cell line transfected Venus transgene by this electroporation was established; that was transgene could be trespassed to the nucleus thought both nucleus and cell membrane (Fig 3E and 3F). It is assumed to be transient pores.

The polarity of interfacial charges switches periodically during droplet bouncing. When the interface is positively charged, negatively charged DNA and cells move toward the interface, while the converse is true for the negatively charged interface. This occurs during movement of the droplet toward the electrode, with induction of the pulsed electric field after the droplet makes contact with the electrode. The distance between the edges was about 6 mm (electrodes for 24-well plates), so the applied electric field intensity was calculated as ≈4.2 kV/cm. However, an intense electric field (> 4.2 kV/cm) would be generated by the “edge” effect. The average frequency of droplet bouncing was about 200 cpm under an applied voltage of 2.5 kV.

Furthermore, application of a more intense but non-lethal electric field to cells can induce an instantaneous short circuit via the extended droplet resulting in effective transfection. However, application of an excessively intense electric field would lead to cell death. Moreover, there is no thermal loading due to the very small electric current, and electrolysis cannot occur. For the above reasons, both high transfection efficiency and cell viability can be achieved using the W/O droplet electroporation method. However, further studies are required to determine the required parameters of the pulsed electric field (strength, pulse width, etc.).

This novel W/O droplet electroporation method has a number of advantages compared with previous transfection techniques: this method allows the simultaneous transfection of various types of DNA into human fibroblast cell lines derived from subjects of various ages, including neuronal cells; both transfection efficiency and cell survival probability are enough high, so transfection can be performed in small numbers of cells in 3-μL droplets. In this study, inclusion of such small numbers of cells (~10000 cells) in droplets was insufficient for fluorescence-activated cell sorting (FACS) analysis.

W/O electroporation is compared with former electroporation, other commercial electroporator, concerning performance. It is in the difference in the principle that W/O electroporation applies a direct current (DC), while a commercial electroporator applies pulsed electric field. Moreover, W/O electroporation applies sample cells in a water-in-oil (W/O) droplet with dielectric silicone oil, while commercial electroporator applies cells in cell suspension. Although the transfection efficiency of electroporation depends on various experimental conditions, such as cell lines, confluency of cells, and pulse parameters, conventional electroporation including commercially available equipment typically shows several tens of percent in transfection efficiency [30]. Therefore the transfection efficiency of our method was not inferior to that of a commercial electroporator.

use of a DC power supply obviates the need for an expensive pulse generator; parallel processing of many wells is possible without marked heat radiation because little electric current flows to the cells with use of a DC power supply; operation can be automated because the processes involved are simple and do not require expert manual intervention; running costs are low because the processes are simple without pulse generator and no expensive chemical reagents are required; and there is little risk of contamination as the sediment of oil droplets can be easily recovered.

Despite these advantages of the W/O droplet electroporation method, a number of problems remain. Although the homogeneous process can be performed in parallel in all wells, some droplets become caught on the electrode and well surface or may be dispersed by burst at the electrical short circuit between electrodes: large droplets attached to both electrodes, form an electrical short-circuit, and burst into many small droplets. All 24-well electrodes for disposable 24-well plates were developed for W/O droplet electroporation (Toyohashi Univ. of Tech.) (S4 Fig). However, multi-well electrodes showed such a wide range of variation in transfection efficiency that some improvement was required to achieve equal efficiency.

Therefore, improved electrode design and the establishment of optimal processing conditions will be necessary to achieve reliable droplet movement in all wells of 96-well plates and automated performance. The distance between electrode edges and the height from the bottom of the well can be changed depending on the volume and viscosity of the dielectric oil to achieve high-frequency reciprocating motion between the electrode pair.

Further development of this W/O droplet electroporation method to facilitate the transport of any charged small molecule, including RNA, peptides, and charged amino acids, across the pores in the cell membrane is required to contribute to genome-wide projects, such as systematic analysis by siRNA, chemical libraries, metabolomics, etc.

Induced pluripotent stem cells (iPS cells) provide new means of developing diagnostic methodologies and novel medicines (i.e., systems in which to check drug effects and side effects or elucidation of the causes of disease), and iPS cell banks for regenerative medicine will be required in future [31, 32]. However, insufficient numbers of iPS cells are currently available for high-throughput screening and regenerative medicine. This W/O droplet electroporation method could be used to efficiently establish iPS cell stocks from patients’ fibroblasts and peripheral tissues cells, transfected with the genes encoding the Yamanaka factors (Oct3/4, Sox2, Klf4, c-Myc).

## Supporting Information

S1 Fig HEK cells transfected after W/O droplet electroporation. Two fluorescence images of HEK cells transfected 4 days after W/O droplet electroporation at 1.8 kV for 5 minutes. Fluorescence signals in each cell were divided by the cell area to determine the mean intensity. Negative control data were average signals from cells without electroporation. Mean intensity of each cell was plotted against cell number in the same field. The red threshold line shows the autofluorescent signal plus 2S.D. value of cells, which is approximately equivalent to 20% of the maximum signal in the same field. The cells with higher fluorescence above the threshold signal were considered to be transfected cells.
(PDF)

S2 Fig Mean intensity against number of days after W/O droplet electroporation. Mean intensity of HEK cells 1, 4, 7, and 12 days after W/O droplet electroporation at 1.8 kV for 5 minutes and of negative controls. Mean intensity was plotted against number of days after W/O droplet electroporation. Data are means ± standard deviation (s.d.) of more than 10 cells counted from at least four different fields on fluorescence microscopy images (**P < 0.0001; unpaired, two-tailed Student’s t test).
(PDF)

S3 Fig Images of hippocampus primary neural cell. Images of hippocampus primary neural cell successfully double transfected with Venus and mCherry (red FP) by W/O droplet electroporation for 5 minutes. Scale bars, 30 μm.
(PDF)

S4 Fig W/O droplet electroporation electrodes for 24-well plates. The droplet actuation device with improved W/O droplet electroporation electrodes for all wells of disposable 24-well plates. Bouncing of water-in-oil droplets was achieved in all wells.
(PDF)

S5 Fig The uptake of YO-PRO 1 measured after the droplet manipulation. Aliquots of 3 μL of the prepared HEK cell suspension containing 10,000 cells with 1 μM YO-PRO 1 were added to the oil, and a DC high voltage (2.0 or 3.0 kV) was applied. Transient pore formation of cell membrane induce YO-PRO 1 (YP) uptakeby W/O droplet electroporation. Fluorescent signal of cells stained with YO-PRO 1 were measured. Statistical analysis was performed using Student's *t*-test. Statistical significance was recognized at * *p* < 0.05, ** *p* < 0.01.
(PDF)

S1 Movie W/O droplet electroporation was performed under a DC electric field intensity of approximately 2.5 kV/cm with electrodes for disposable 96-well plates. The droplets of PBS buffer were colored by trypan blue without cells and plasmid DNA.
(AVI)

## References

[1] Chang DC, Chassy BM, Saunder JA, Sowers AE (1992) Guide to Electroporation and Electrofusion. Academic, San Diego, CA, USA.

[2] Gehl J (2003) Electroporation: theory and methods, perspectives for drug delivery, gene therapy and research. Acta Physiol Scand 177: 437–447. 12648161 10.1046/j.1365-201X.2003.01093.x

[3] Lin YC, Huang MY (2001) Electroporation microchips for in vitro gene transfection. Journal of Micromechanics and Microengineering 11: 542–547.

[4] Lu H, Schmidt MA, Jensen KF (2005) A microfluidic electroporation device for cell lysis. Lab on a Chip 5: 23–29. 15616736 10.1039/b406205a

[5] Fox MB, Esveld DC, Valero A, Luttge R, Mastwijk HC, Bartels PV et al (2006) Electroporation of cells in microfluidic devices: a review. Analytical and Bioanalytical Chemistry 385: 474–485. 16534574 10.1007/s00216-006-0327-3

[6] Kim SK, Kim JY, Kim KP, Chung TD (2007) Continuous low-voltage dc electroporation on a microfluidic chip with polyelectrolytic salt bridges. Analytical Chemistry 79: 7761–7766. 17874852 10.1021/ac071197h

[7] Geng T, Zhan Y, Wang HY, Witting SR, Cornetta KG, Lu C. et al (2010) Flow-through electroporation based on constant voltage for large-volume transfection of cells. Journal of Controlled Release 144: 91–100. 10.1016/j.jconrel.2010.01.030 20117155

[8] Adamo A, Arione A, Sharei A, Jensen KF (2013) Flow-Through Comb Electroporation Device for Delivery of Macromolecules. Analytical Chemistry 85: 1637–1641. 10.1021/ac302887a 23259401 PMC3565052

[9] Wang SN, Lee LJ (2013) Micro-/nanofluidics based cell electroporation. Biomicrofluidics 7: 11301 10.1063/1.4774071 23405056 PMC3555966

[10] Luo C, Yang X, Fu Q, Sun M, Ouyang Q, Chen Y, et al (2006) Picoliter-volume aqueous droplets in oil: Electrochemical detection and yeast electroporation. Electrophoresis 27:1977–1983. 16596709 10.1002/elps.200500665

[11] Zhan YH, Wang J, Bao N, Lu C (2009) Electroporation of Cells in Microfluidic Droplets. Analytical Chemistry 81: 2027–2031. 10.1021/ac9001172 19199389

[12] Xiao K, Zhang M, Chen S, Wang L, Chang DC, Wen W., et al (2010) Electroporation of micro-droplet encapsulated HeLa cells in oil phase. Electrophoresis 31: 3175–3180. 10.1002/elps.201000155 20803502

[13] Chen F, Zhan Y, Geng T, Lian H, Xu P, Lu C., et al (2011) Chemical Transfection of Cells in Picoliter Aqueous Droplets in Fluorocarbon Oil. Analytical Chemistry 83: 8816–8820. 10.1021/ac2022794 21967571

[14] Hase M, Watanabe SN, Yoshikawa K (2006) Rhythmic motion of a droplet under a dc electric field. Physical Review E 74(4). 10.1103/PhysRevE.74.046301 17155167

[15] Jung Y-M, Oh H-C, Kang IS (2008) Electrical charging of a conducting water droplet in a dielectric fluid on the electrode surface. Journal of Colloid and Interface Science 322: 617–623. 10.1016/j.jcis.2008.04.019 18442825

[16] Khorshidi B, Jalaal M, Esmaeilzadeh E, Mohammadi F (2010) Characteristics of deformation and electrical charging of large water drops immersed in an insulating liquid on the electrode surface. Journal of Colloid and Interface Science 352: 211–220. 10.1016/j.jcis.2010.08.026 20822774

[17] Takinoue M, Atsumi Y, Yoshikawa K (2010) Rotary motion driven by a direct current electric field. Applied Physics Letters 96: 104105.

[18] Hamlin BS, Ristenpart WD (2012) Transient reduction of the drag coefficient of charged droplets via the convective reversal of stagnant caps. Physics of Fluids 24: 012101.

[19] Im do J, Ahn MM, Yoo BS, Moon D, Lee DW, Kang IS., et al (2012) Discrete Electrostatic Charge Transfer by the Electrophoresis of a Charged Droplet in a Dielectric Liquid. Langmuir 28: 11656–11661. 10.1021/la3014392 22846106

[20] Lee DW, Im DJ, Kang IS (2012) Electrophoretic motion of a charged water droplet near an oil-air interface. Applied Physics Letters 100: 221602.

[21] Wang QM, Suo ZG, Zhao XH (2012) Bursting drops in solid dielectrics caused by high voltages. Nature Communications 3: 1157–63. 10.1038/ncomms2178 23093194 PMC3681824

[22] Im do J, Noh J, Yi NW, Park J, Kang IS. (2011) Influences of electric field on living cells in a charged water-in-oil droplet under electrophoretic actuation. Biomicrofluidics 5: 044112–10. 10.1063/1.3665222 PMC3364810 22662063

[23] Asada A, Aoki H, Kurita H, Antoniu A, Yasuda H, Takashima K. et al (2013) A Novel Gene Transformation Technique Using Water-in-Oil Droplet in an Electrostatic Field. IEEE Transactions on Industry Applications 49: 311–315.

[24] Nagai T, Sawano A, Park ES, Miyawaki A (2001) Circularly permuted green fluorescent proteins engineered to sense Ca^2+^ . P Natl Acad Sci USA 98: 3197–3202. 10.1073/pnas.051636098 PMC30630 11248055

[25] Yamazaki S, Numano R, Abe M, Hida A, Takahashi R, Ueda M, et al (2000) Resetting central and peripheral circadian oscillators in transgenic rats. Science 288: 682–685. 10784453 10.1126/science.288.5466.682

[26] Kawaguchi S, Shinozaki A, Obinata M, Saigo K, Sakaki Y, Tei H., et al (2007) Establishment of cell lines derived from the rat suprachiasmatic nucleus. Biochem Bioph Res Co 355: 555–561 10.1016/j.bbrc.2007.02.009 17306763

[27] Pucihar G, Kotnik T, Miklavcic D, Teissie J. (2008) Kinetics of transmembrane transport of small molecules into electropermeabilized cells. Biophysical journal 95:2837–2848. 10.1529/biophysj.108.135541 18539632 PMC2527253

[28] Pakhomov AG, Gianulis E, Vernier PT, Semenov I, Xiao S, Pakhomova ON. Multiple (2015) Nanosecond electric pulses increase the number but not the size of long-lived nanopores in the cell membrane. Biochimica et biophysica acta.;1848:958–966. 10.1016/j.bbamem.2014.12.026 25585279 PMC4331219

[29] Gianulis EC, Lee J, Jiang C, Xiao S, Ibey BL, Pakhomov AG. (2015) Electroporation of mammalian cells by nanosecond electric field oscillations and its inhibition by the electric field reversal. Scientific reports. 5:13818 10.1038/srep13818 26348662 PMC4562301

[30] Knight JS, Sharma N, Kalman DE, Robertson ES. (2004) A cyclin-binding motif within the amino-terminal homology domain of EBNA3C binds cyclin A and modulates cyclin A-dependent kinase activity in Epstein-Barr virus-infected cells. J Virol. 78:12857–12867. 15542638 10.1128/JVI.78.23.12857-12867.2004 PMC524968

[31] Takahashi K, Tanabe K, Ohnuki M, Narita M, Ichisaka T, Tomoda K, et al (2007) Induction of pluripotent stem cells from adult human fibroblasts by defined factors. Cell 131: 861–872 18035408 10.1016/j.cell.2007.11.019

[32] Takahashi K, Yamanaka S (2006) Induction of pluripotent stem cells from mouse embryonic and adult fibroblast cultures by defined factors. Cell 126: 663–676 16904174 10.1016/j.cell.2006.07.024

