# Supplementary material for: Novel Parallelized Electroporation by Electrostatic Manipulation of a Water-in-Oil Droplet as a Microreactor
Source: PLoS One. 2015 Dec 9;10(12):e0144254. doi: 10.1371/journal.pone.0144254 (PMC4674099; doi:10.1371/journal.pone.0144254)
Supplement: S1 Fig — Two fluorescence images of HEK cells transfected 4 days after W/O droplet electroporation at 1.8 kV for 5 minutes. Fluorescence signals in each cell were divided by the cell area to determine the mean intensity. Negative control data were average signals from cells without electroporation. Mean intensity of each cell was plotted against cell number in the same field. The red threshold line shows the autofluorescent signal plus 2S.D. value of cells, which is approximately equivalent to 20% of the maximum signal in the same field. The cells with higher fluorescence above the threshold signal were considered to be transfected cells. (PDF) [file pone.0144254.s001.pdf]

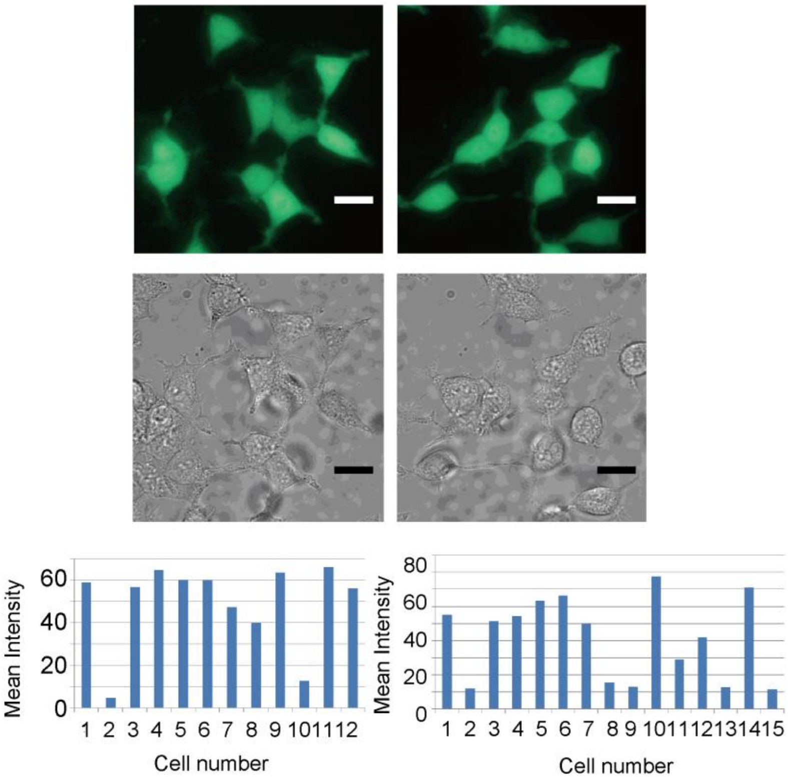

S1 Fig. Two fluorescence images of HEK cells transfected 4 days after W/O droplet electroporation and the mean intensity of each cell. Two fluorescence images of HEK cells transfected 4 days after W/O droplet electroporation at 1.8 kV for 5 minutes. Fluorescence signals in each cell were divided by the cell area to determine the mean intensity. Negative control data were average signals from cells without electroporation. Mean intensity of each cell was plotted against cell number in the same field. The red threshold line shows the autofluorescent signal plus 2S.D. value of cells, which is approximately equivalent to 20% of the maximum signal in the same field. The cells with higher fluorescence above the threshold signal were considered to be transfected cells.

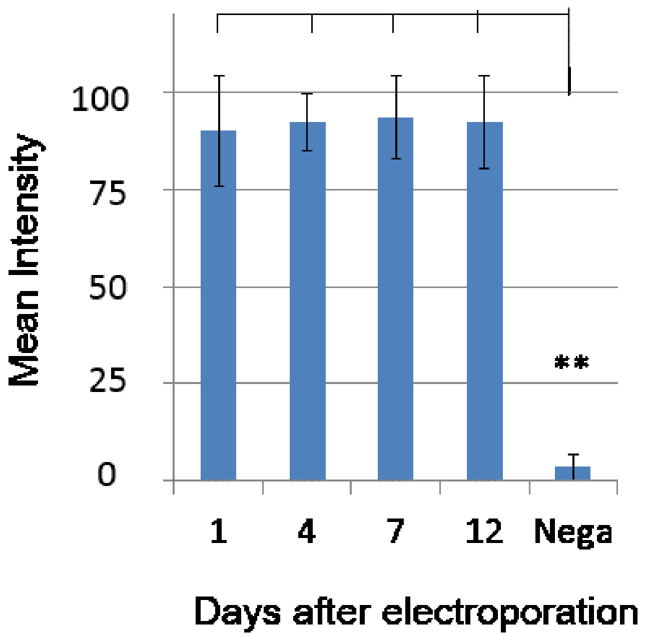

S2 Fig. The mean intensity of HEK cells 1, 4, 7, and 12 days after W/O droplet electroporation and negative controls. The mean intensity of HEK cells 1, 4, 7, and 12 days after W/O droplet electroporation at 1.8 kV for 5 minutes and of negative controls. Mean intensity was plotted against number of days after W/O droplet electroporation. Data are means ± standard deviation (s.d.) of more than 10 cells counted from at least four different fields on fluorescence microscopy images (***P* < 0.0001; unpaired, two-tailed Student's *t* test).

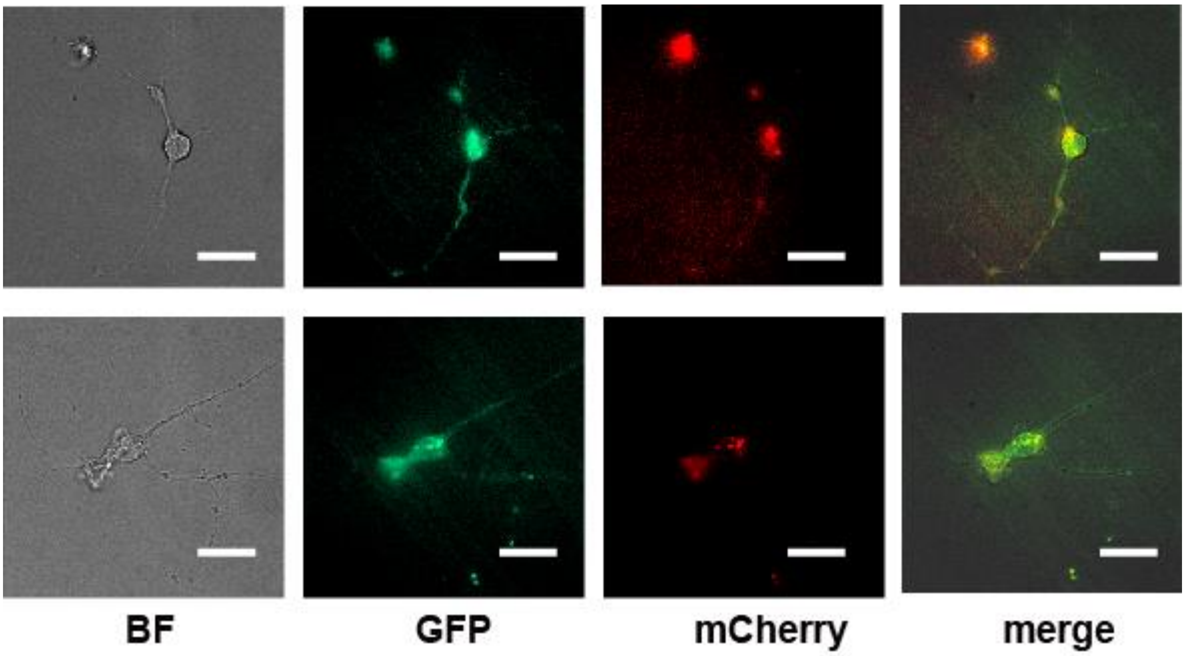

S3 Fig. Images of hippocampus primary neural cell lines successfully double transfection. Images of hippocampus primary neural cell lines successfully double transfected with Venus and mCherry (red FP) by W/O droplet electroporation for 5 minutes. Scale bars, 30 µm.

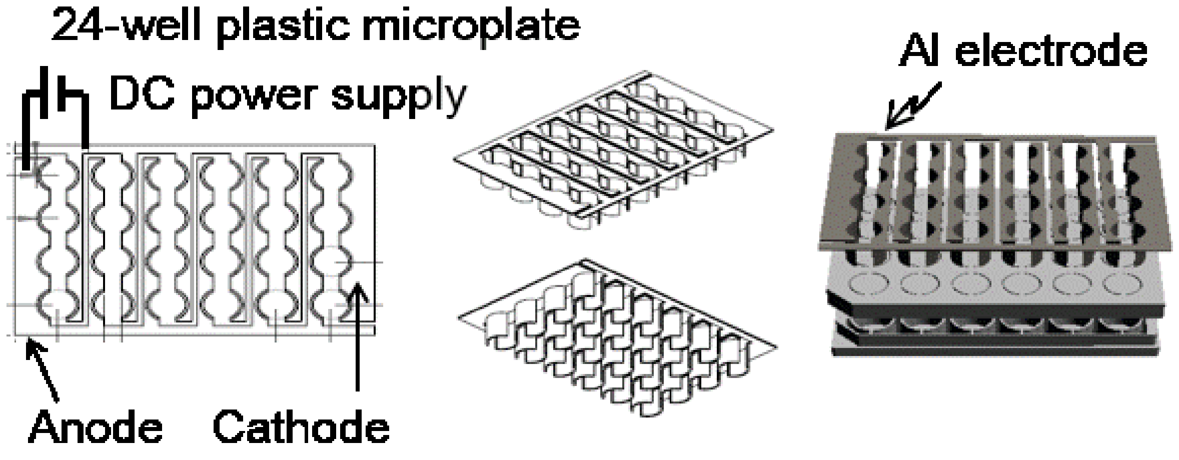

S4 Fig. The W/O droplet electroporation electrodes of disposable 24-well plates. The droplet actuation device with improved W/O droplet electroporation electrodes for all wells of disposable 24-well plates. Bouncing of water-in-oil droplets was achieved in all wells.

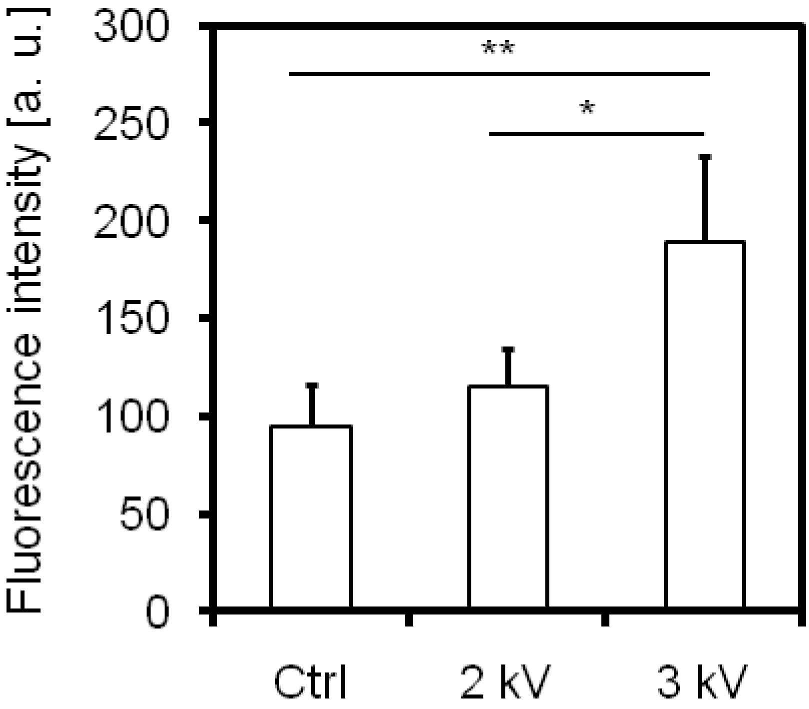

S5 Fig. The uptake of YO-PRO 1 measured after the droplet manipulation. Aliquots of 3 μL of the prepared HEK cell suspension containing 10,000 cells with 1 μM YO-PRO 1 were added to the oil, and a DC high voltage (2.0 or 3.0 kV) was applied. YO-PRO 1 (YP) passed through cell pass through a tunnel by W/O droplet electroporation. Fluorescent signal of cells by stained YO-PRO 1were measured. Statistical analysis was performed using Student's t-test. Statistical significance was recognized at * $p < 0.05$, ** $p < 0.01$.
