# Supplementary material for: Novel Parallelized Electroporation by Electrostatic Manipulation of a Water-in-Oil Droplet as a Microreactor
Source: PLoS One. 2015 Dec 9;10(12):e0144254. doi: 10.1371/journal.pone.0144254 (PMC4674099; doi:10.1371/journal.pone.0144254)
Supplement: S2 Fig — Mean intensity of HEK cells 1, 4, 7, and 12 days after W/O droplet electroporation at 1.8 kV for 5 minutes and of negative controls. Mean intensity was plotted against number of days after W/O droplet electroporation. Data are means ± standard deviation (s.d.) of more than 10 cells counted from at least four different fields on fluorescence microscopy images (**P < 0.0001; unpaired, two-tailed Student’s t test). (PDF) [file pone.0144254.s002.pdf]

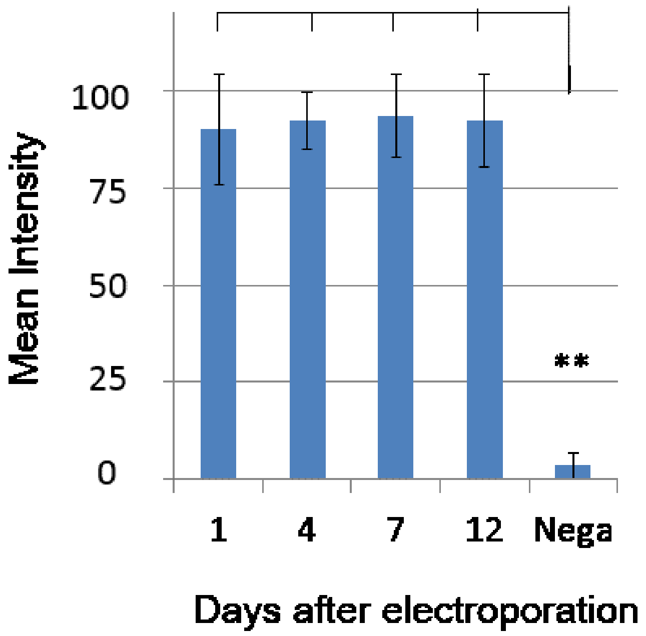

S2 Fig. The mean intensity of HEK cells 1, 4, 7, and 12 days after W/O droplet electroporation and negative controls. The mean intensity of HEK cells 1, 4, 7, and 12 days after W/O droplet electroporation at 1.8 kV for 5 minutes and of negative controls. Mean intensity was plotted against number of days after W/O droplet electroporation. Data are means ± standard deviation (s.d.) of more than 10 cells counted from at least four different fields on fluorescence microscopy images (***P* < 0.0001; unpaired, two-tailed Student's *t* test).
