# Supplementary figures and images for: Novel Parallelized Electroporation by Electrostatic Manipulation of a Water-in-Oil Droplet as a Microreactor
Source: PLoS One. 2015 Dec 9;10(12):e0144254. doi: 10.1371/journal.pone.0144254 (PMC4674099; doi:10.1371/journal.pone.0144254)

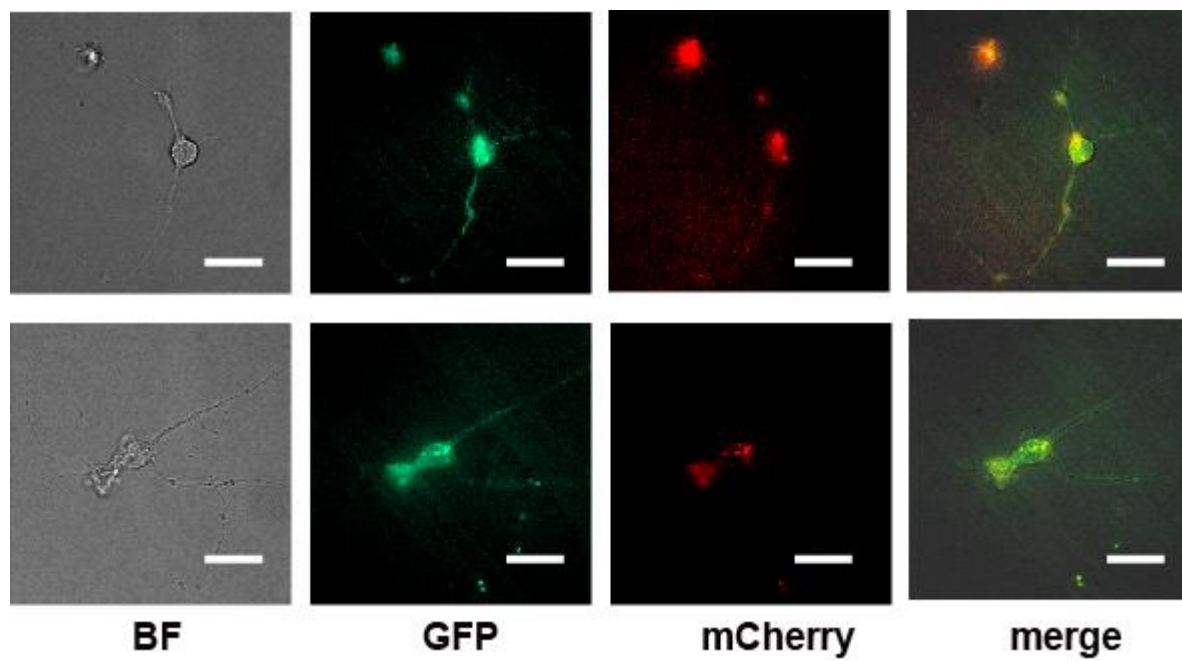

Supplement: S3 Fig — Images of hippocampus primary neural cell successfully double transfected with Venus and mCherry (red FP) by W/O droplet electroporation for 5 minutes. Scale bars, 30 μm. (PDF) [file pone.0144254.s003.pdf]

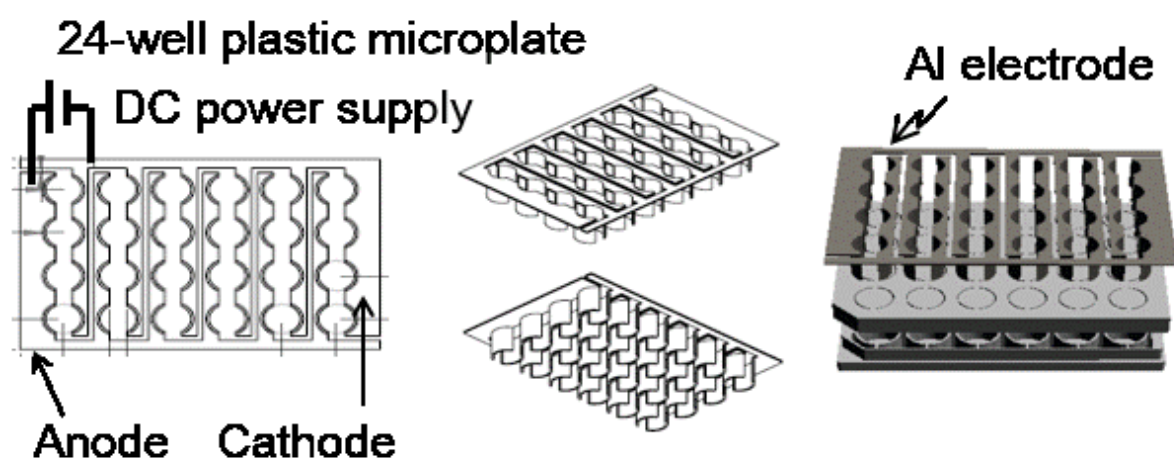

Supplement: S4 Fig — The droplet actuation device with improved W/O droplet electroporation electrodes for all wells of disposable 24-well plates. Bouncing of water-in-oil droplets was achieved in all wells. (PDF) [file pone.0144254.s004.pdf]

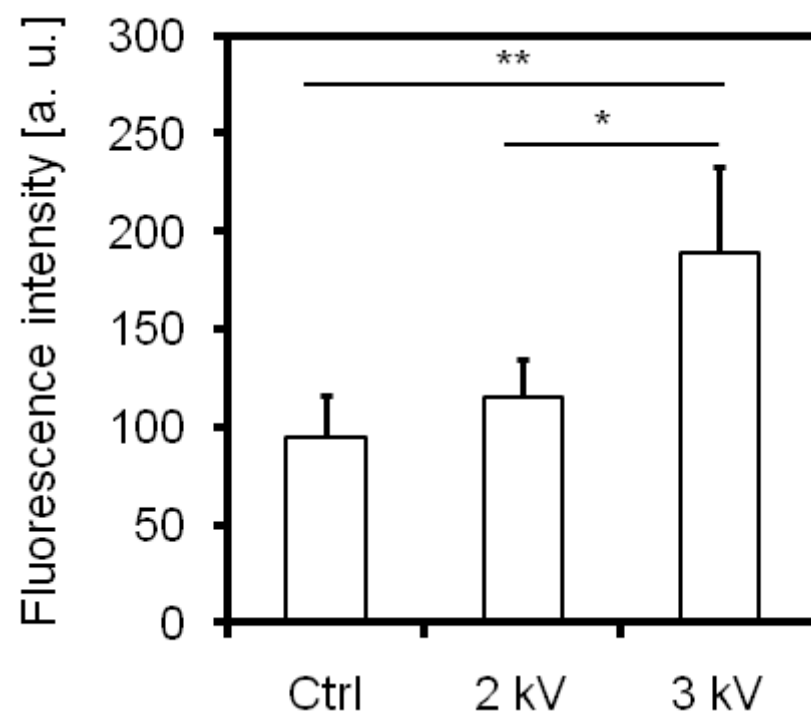

Supplement: S5 Fig — Aliquots of 3 μL of the prepared HEK cell suspension containing 10,000 cells with 1 μM YO-PRO 1 were added to the oil, and a DC high voltage (2.0 or 3.0 kV) was applied. Transient pore formation of cell membrane induce YO-PRO 1 (YP) uptakeby W/O droplet electroporation. Fluorescent signal of cells stained with YO-PRO 1 were measured. Statistical analysis was performed using Student's t-test. Statistical significance was recognized at * p < 0.05, ** p < 0.01. (PDF) [file pone.0144254.s005.pdf]
